# Impact of helminth co-infection and treatment on mycobacterial growth inhibition in UK migrants with TB infection

**DOI:** 10.5588/ijtldopen.24.0528

**Published:** 2025-04-09

**Authors:** S. Anwar, C.F. Turienzo, L. Tsang, S.G. Smith, H. Fletcher, F. Toulza, J.M. Cliff, M. Brown, H.M. Dockrell

**Affiliations:** ^1^ Department of Infection Biology, Faculty of Infectious and Tropical Diseases, London School of Hygiene & Tropical Medicine, London, UK;; ^2^ Department of Microbiology & Immunology, Bangabandhu Sheikh Mujib Medical University, Dhaka, Bangladesh;; ^3^ University College London Hospital, London, UK;; ^4^ King’s College London, London, UK;; ^5^ The North West London Hospital NHS Trust, Northwick Park Hospital, London, UK;; ^6^ Brunel University, London, UK;; ^7^ Johnson&Johnson, London, UK;; ^8^ Guys and St Thomas NHS Trust, London, UK;; ^9^ Department of Clinical Research, London School of Hygiene & Tropical Medicine, London, UK.

**Keywords:** tuberculosis, mycobacterial growth inhibition assay, anti-helminthic treatment, migration

## Abstract

**BACKGROUND:**

TB and helminth infections are co-endemic in many parts of the world. This has led to the hypothesis that immunomodulation due to helminth infections could adversely affect the ability to control *Mycobacterium tuberculosis* infection. Anti-helminthic treatment has been associated with improved anti-mycobacterial cellular responses and decreases in the frequency of regulatory T-cells. We therefore investigated how control of mycobacterial growth and anti-mycobacterial immune responses are modulated in helminth and TB co-infected individuals using a mycobacterial growth inhibition assay (MGIA).

**METHODS:**

Migrants with eosinophilia or suspected/diagnosed helminth infection and/or TB infection (TBI) were recruited when attending University College London Hospitals (London, UK) and followed up after completing anti-helminthic treatment. Mycobacterial growth inhibition was assessed using the BACTEC™ MGIT™ system after 72 hours of co-culture of peripheral blood mononuclear cells (PBMC) with *M*. *bovis* bacille Calmette-Guérin (BCG) or *M. tuberculosis* Erdman.

**RESULTS:**

Anti-helminthic treatment reduced total and helminth-specific antibodies in helminth-infected and TBI–helminth co-infected individuals. Helminth-infected individuals displayed lower growth inhibition in the MGIA than those without helminth infections, and mycobacterial growth inhibition improved after anti-helminthic treatment. Blocking interleukin-10 (IL-10) and transforming growth factor-beta (TGF-β) improved mycobacterial growth inhibition, while blocking interferon-gamma (IFN-γ) did not alter growth inhibition.

**CONCLUSION:**

Infection with helminths such as *Schistosoma mansoni* and *Strongyloides* spp. may reduce the ability to control mycobacterial growth.

TB and helminth infections coincide in many parts of the world, which has led to the hypothesis that concomitant helminth infections could affect anti-mycobacterial immunity. By creating an anti-inflammatory environment, helminth co-infection might dampen protective and immunopathological responses to *Mycobacterium tuberculosis*. Helminth-induced T-helper 2 (Th2) and regulatory T-cell (Treg) responses may contribute to depressed *M. tuberculosis*-specific Th1 responses.^1^ Interleukin 10 (IL-10) and transforming growth factor-beta (TGF-β) cytokine production during chronic helminth infection may also mediate antigen-specific T-cell hypo-responsiveness, including through impaired signal transduction.^2^

Mycobacterial growth inhibition assays (MGIAs) are functional assays in which the ability of ex vivo blood samples or peripheral blood mononuclear cells (PBMC) is tested for their ability to inhibit the growth of mycobacteria.^3–5^ Recently improved protocols for MGIA using the commercial BACTEC™ MGIT™ system (BD, Franklin Lakes, NJ, USA) have been developed,^6^ and used to assess the impact of infection or vaccination on growth inhibition. Helminth infections appear to play an important role in modulating the immune response in TB.^1^ Co-infection with helminths may contribute to the poor outcome of *M. tuberculosis* infection by skewing the immune response to the production of immunoglobulin (Ig) E antibodies, which is IL-4 dependent.^7^ Adjobimey et al. showed that high plasma concentrations of IgG4 in patients with filaria were associated with hypo-responsiveness, high parasite load and high plasma levels of IL-10 and TGF-β.^8^

In a UK cohort of migrants co-infected with helminths and *M. tuberculosis*, we previously showed reduced CD4^+^ T cell interferon-gamma (IFN-γ) responses to *M. tuberculosis* purified protein derivative (PPD) and early secreted antigen-6/culture filtrate protein-10 (ESAT-6/CFP10) and secretion of IFN-γ, tumour necrosis factor (TNF) and IL-2 from PBMC, which reversed after anti-helminthic treatment.^9^ We have now explored the potential impact of helminth infection on the control of mycobacterial growth of *M. tuberculosis* by measuring mycobacterial growth inhibition in migrants living in London, UK, with TB infection (TBI) and with or without helminth infection (*Strongyloides* or *Schistosoma*) using MGIA and whether anti-helminthic treatment modulates these immune responses. We also analysed IgE and IgG4 responses to characterise the immune response and effect of treatment further. Since ongoing exposure or re-infection following anti-helminthic treatment would not occur in the United Kingdom, this study provided a unique opportunity to assess the effect of anti-helminthic treatment in helminth-infected individuals. Our data showed that helminth infection reduced mycobacterial growth inhibition, which improved after anti-helminthic treatment, indicating that the modulation of the immune response is helminth-mediated.

## MATERIALS AND METHODS

### Study design, population and samples

We conducted a longitudinal prospective cohort study in London, UK. Migrants from endemic countries with eosinophilia or suspected/diagnosed helminth infection and with or without TBI were recruited between January 2014 and September 2016 at the Hospital of Tropical Diseases (HTD) and TB clinics at University College London Hospitals (UCLH). This ambulatory cohort did not recruit inpatient or acutely unwell patients. We excluded migrants aged <18 years, with known HIV infection or immunosuppressive conditions, with microbiological or radiological evidence of TB disease, or with previous or ongoing treatment for TBI or helminth infection. The control group consisted of individuals with a history of living in tropical countries who attended the clinic to investigate non-specific symptoms of asymptomatic eosinophilia and were found to be negative for helminth infection and TBI. Sample size calculations for recruitment to the parent IDEA study were based on consensus predictions of the prevalence of TBI and local evidence of the prevalence of diagnosed helminth infection among patients with eosinophilia, sufficient to generate enough patients with complete time points for immunological assays for regulatory T-cells. Patients recruited for this study were consecutive patients for whom all time points were available. Demographic information on the individuals studied is given in the Table. The largest number of individuals recruited in all groups came from Bangladesh, followed by Nigeria, with smaller numbers of individuals from India, The Philippines, Kenya, Ghana, Zimbabwe, Somalia, Eritrea, Ethiopia, Pakistan, South Africa and Sierra Leone.

**Table.** Characteristics of study participants.

Variable	Controls (*n* = 32)	TBI (*n* = 32)	Helminth infected (*n* = 36)	TBI-helminth infected (*n* = 16)
Male, *n* (%)	13 (40.6)	18 (56.3)	18 (50)	13 (81.2)
Female, *n* (%)	19 (59.4)	14 (43.7)	18 (50)	3 (18.8)
Mean age (years)	37.8	37.5	47.4	54.6
Time spent in UK (years, range)	4–49	2–33	2–46	6–48
Strongyloides infection	0	0	15	9
Schistosoma infection	0	0	16	7
Other helminths	0	0	5	0

TBI = TB-infected.

TBI was diagnosed using a QuantiFERON^®^-TB Gold (QFT-G; Qiagen, Hilden, Germany) interferon-gamma release assay (IGRA) in the absence of microbiological and radiological evidence of TB disease.^9^ The QuantiFERON-TB Gold in Tube (QFT-GIT) assay performed on all those recruited involves collecting 1 ml of whole blood into specialised tubes containing three specific antigens: Nil (negative control), TB1, and TB2 (Mtb-specific antigens), a Mitogen tube (positive control). The blood samples were mixed gently and incubated at 37°C for 16–24 h to allow immune cells to react with the antigens. After incubation, the tubes were centrifuged to separate the plasma, which was then analysed for IFN-γ release using an enzyme-linked immunosorbent assay (ELISA). Optical density values were measured using an ELISA reader. The QFT Analysis Software was used to analyse raw data and to calculate results. For the QFT-GIT test to be valid, the nil value must be less than or equal to 8.0 IU/mL, and the mitogen value (positive control for IFN-γ production) must be at least 0.5 IU/mL higher than the nil value. The QFT-GIT test is considered positive if the IFN-γ response if the TB antigen minus nil value is at least 0.35 IU/mL. The tests were performed by the routine diagnostic laboratory at UCH.

All the individuals recruited for this study were tested for helminths. A single stool sample was collected for helminth diagnosis using microscopy, and strongyloides charcoal culture and serum samples were tested for anti-helminth antibodies, which was in line with the national guidance for the investigation of eosinophilia.

For diagnosis of *Strongyloides* spp., serum samples were analysed by a commercial ELISA for anti-*Strongyloides* antibodies (using *S. ratti* somatic larval antigen) according to the manufacturer’s protocol using a cut-off based on a low positive serum (Bordier, Paris, France). The assay has a sensitivity of 88% and specificity of 100%. An in-house ELISA assay (the Hospital for Tropical Diseases (HTD) is the UK national reference laboratory for parasitology) was performed to detect IgG antibodies to *Schistosoma* egg antigens. The TBI-helminth co-infected group comprised individuals who were QFT positive (with no evidence of active TB disease) and had microscopy- or serology-confirmed evidence of helminth infection.

At recruitment (Visit 1, V1), participants provided serological samples for parasitological and TBI diagnosis (unless results were already available). Participants were classified into four groups: both helminth and TBI-negative (controls); helminth-negative, TBI-positive; helminth-positive, TBI-negative; and both helminth and TBI-positive. Study participants with helminths and/or TBI were managed according to current HTD, Public Health England, and National Institute for Health and Care Excellence (NICE) guidelines.^10^ Patients with TBI without evidence of active disease were referred for treatment of latent TBI according to contemporaneous NICE guidance *Tuberculosis - NCBI Bookshelf,* which recommended treatment in high-risk groups (not including DM) and those aged >35 years. This has been subsequently updated. Helminth infections were treated using current HTD guidelines (ivermectin 200 mcg/kg twice for *Strongyloides*; praziquantel 40 mg/kg once for *Schistosomiasis*; and albendazole or praziquantel as appropriate for other species, provided in the on-site clinic pharmacy on day of attendance). At least 3 months after recruitment, study participants returned for a follow-up visit (V2) when a second QFT test was performed and blood collected for further immunological assays. Participants in the control and TBI-only groups without intervention also returned for a follow-up visit.

### Quantitation of total and specific IgG4 and IgE antibodies by ELISA

Total IgG4 and IgE concentrations were measured using ELISA kits according to the manufacturer’s instructions (Invitrogen, Waltham, MA, USA; #88-50590 for IgG4 and #88-60610 for IgE) with detection limits of 31.3 ng/ml IgG4 and 7.8 ng/ml IgE. To detect specific antibodies, 96-well ELISA plates (Corning™, New York, NY, USA) were coated with 100 μl of PPD (Statens Serum Institute, Copenhagen, Denmark) at 10 µg/mL or *S. mansoni* soluble egg antigen (SEA) kindly provided by Dr H Smits, Leiden University Medical Centre, at 20 µg/ml and incubated overnight at 4°C. After washing with PBS containing 0.05% Tween-20 (v/v), plates were blocked for 2 h at room temperature with 250 μl blocking buffer and washed with wash buffer four times. Hundred microlitres of each diluted test plasma sample and positive and negative controls (diluted 1 in 10, 1 in 100 and 1 in 1,000 in PBS with 1% Tween 20 and 10% BSA) were added in triplicate. Plates were incubated for 2 h at room temperature on a microplate shaker at 400 rpm. After washing, 100 μl of pre-titrated horseradish peroxidase (HRP) conjugated anti-human IgE monoclonal detection antibody was added to each well for 1 h at room temperature on a microplate shaker at 400 rpm. About 100 μl 3,3’,5,5’-tetramethyl benzidine (TMB) (Becton Dickinson) was added to each well and incubated at room temperature for 15 min. The reaction was stopped by adding 100 μl of 2M sulphuric acid (Sigma-Aldrich, St Louis, MO, USA) per well. Absorbance was measured using a microplate reader at 450 nm.

### Mycobacterial growth inhibition assay

PBMCs were isolated by density centrifugation on histopaque (Sigma-Aldrich UK, Gillingham, UK), frozen with 20% fetal bovine serum (FBS; Invitrogen) and 10% dimethyl sulphoxide (DMSO) (Sigma-Aldrich) and stored at –80° C for 24 h before transfer to liquid nitrogen and were then tested within 3 months of storage. The viability of the thawed PBMC was >95%. Thawed PBMC were incubated overnight at 37°C with CO_2_ at 1 x 10^6^ cells/ml in MGIT (Mycobacterial Growth Inhibition Tube) medium. Cells were counted, washed and re-suspended in MGIT medium at 1 x 10^6^ PBMC/300 µl medium for 2 h at 37°C in RPMI 1640 containing 10 units/ml of benzonase (Novagen, Darmstadt, Germany), washed and re-suspended in RPMI 1640 with 25 mM HEPES (Sigma) with 2 mM L-glutamine and 10% filtered, heat-inactivated, pooled human AB serum (Sigma). PBMC (1 x 10^6^ per tube) was added to 2 ml screw-cap microtubes (Sarstedt, Nümbrecht, Germany) with a pre-determined, optimal quantity of bacille Calmette-Guérin (BCG Pasteur strain supplied by Aeras, Rockville, MD, USA), 30 colony-forming unit (CFU) from a stock concentration of 1 x 10^8^ CFU/ml or *M. tuberculosis* Erdman 30 CFU from a stock concentration of 1 x 10^8^ CFU/ml and made up to a final volume of 600 µl. Tubes were incubated at 37°C with 360° rotation for 96 h. Following incubation, the cells and remaining BCG were pelleted by centrifugation at 15,300 g, and cells were lysed by incubation in sterile water with vortexing. The contents of a single tube were then transferred into an MGIT tube, and time-to-positivity (TTP) was determined using an MGIT 960 machine (Becton Dickinson). On Day 0, duplicate direct-to-MGIT viability control tubes were set up by directly inoculating supplemented BACTEC MGIT tubes with 300 µl of BCG-containing 30 CFU, as used in the test tubes.

To assess the effect of adding recombinant cytokines to the MGIAs, recombinant human IL-10 (BioLegend, San Diego, CA, USA; #571002) and TGF-β (BioLegend, #580702) were added to the MGIA tube at time 0. To block cytokines, monoclonal antibodies to human IFN-γ ((BioLegend, #502501), human IL-10 (BioLegend, #506802) or human TGF-β (BioLegend, #525301) were added at time 0.

### Ethical statement

Regulatory approvals were obtained from the NHS Research Ethics Committee (Ref. 11/H0713/12), the Health Research Authority (HRA) and the Ethics Committee of the London School of Hygiene & Tropical Medicine (LSHTM Ethics Ref No 7,758). All participants signed written informed consent.

## RESULTS

### Study population

A total of 155 individuals were enrolled in London: 32 had TBI, 36 had helminth infection, 16 had both TBI and helminth infection, and 32 were uninfected controls. The rest of the 39 controls were investigated for helminth infections and found not to have helminth infections other than Schistosomiasis and Strongyloidiasis. Demographic information on the individuals studied is given in the Table. The control group consisted of individuals with a history of living in tropical countries who attended the clinic with gastrointestinal or respiratory symptoms but who were negative for both helminth infection and TBI; active TB disease was excluded in those with TBI. Most of the patients recruited in the study were from Bangladesh, Nigeria, India, Kenya, Ghana, Zimbabwe, and Somalia. Time spent in the UK ranged from a few years to >20 years.

The time intervals between V1 and V2 were a median of 4 months (2–7 months) for controls, 6 months (3–9 months) for TBI, 4 months (3–9 months) for helminth-infected patients and 6 months (4–9 months) for the TBI-helminth co-infected patients. Of the helminth-infected group, 41.6% of the patients presented with Strongyloidiasis, 44.4% with Schistosomiasis and 13.8% with tapeworm. Of the TBI-helminth co-infected individuals, 56.3% presented with Strongyloidiasis, and 43.7% with Schistosomiasis.

### IgG4 and IgE antibody concentrations in plasma and changes following anti-helminthic treatment

**Figure 1.**
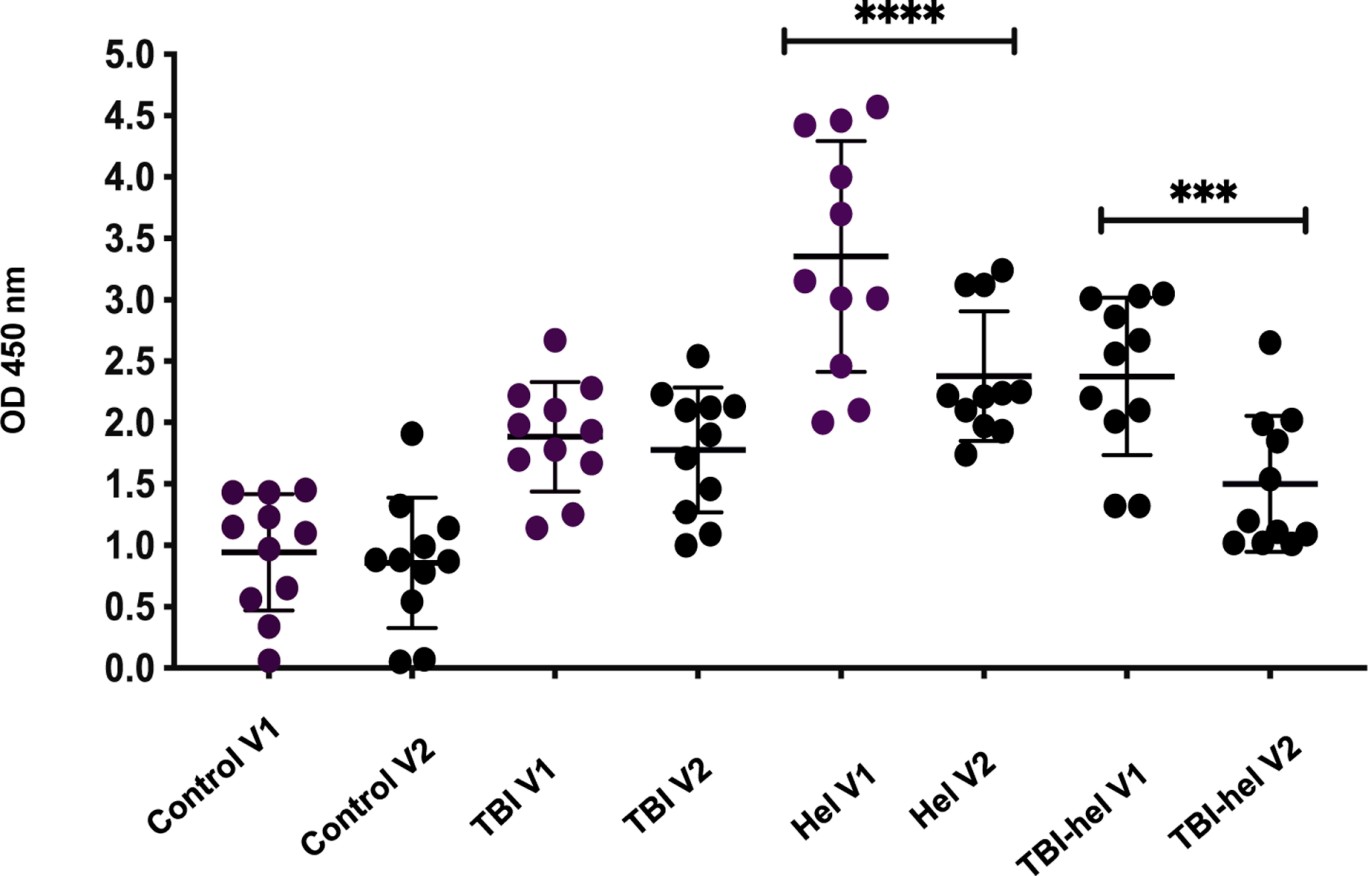
Antigen-specific IgE responses were measured using ELISA in controls, TBI, helminth-infected patients and TBI-helminth co-infected patients. Plasma from 11 patients of each group were used in the ELISA. Helminth-infected patients were infected with *S.mansoni*, and *S.mansoni* SEA antigen was used as antigen. OD values at 450 nm were measured. Bars represent means with SD. A repeated measures ANOVA was performed, where *** represented a *P* < 0.005 and **** represented a *P* < 0.000. OD = optical density; V1 = Visit 1; TBI = TB infection; ELISA = enzyme-linked immunosorbent assay; SEA = *S. mansoni* soluble egg antigen; SD = standard deviation; ANOVA = analysis of variance.

**Figure 2.**
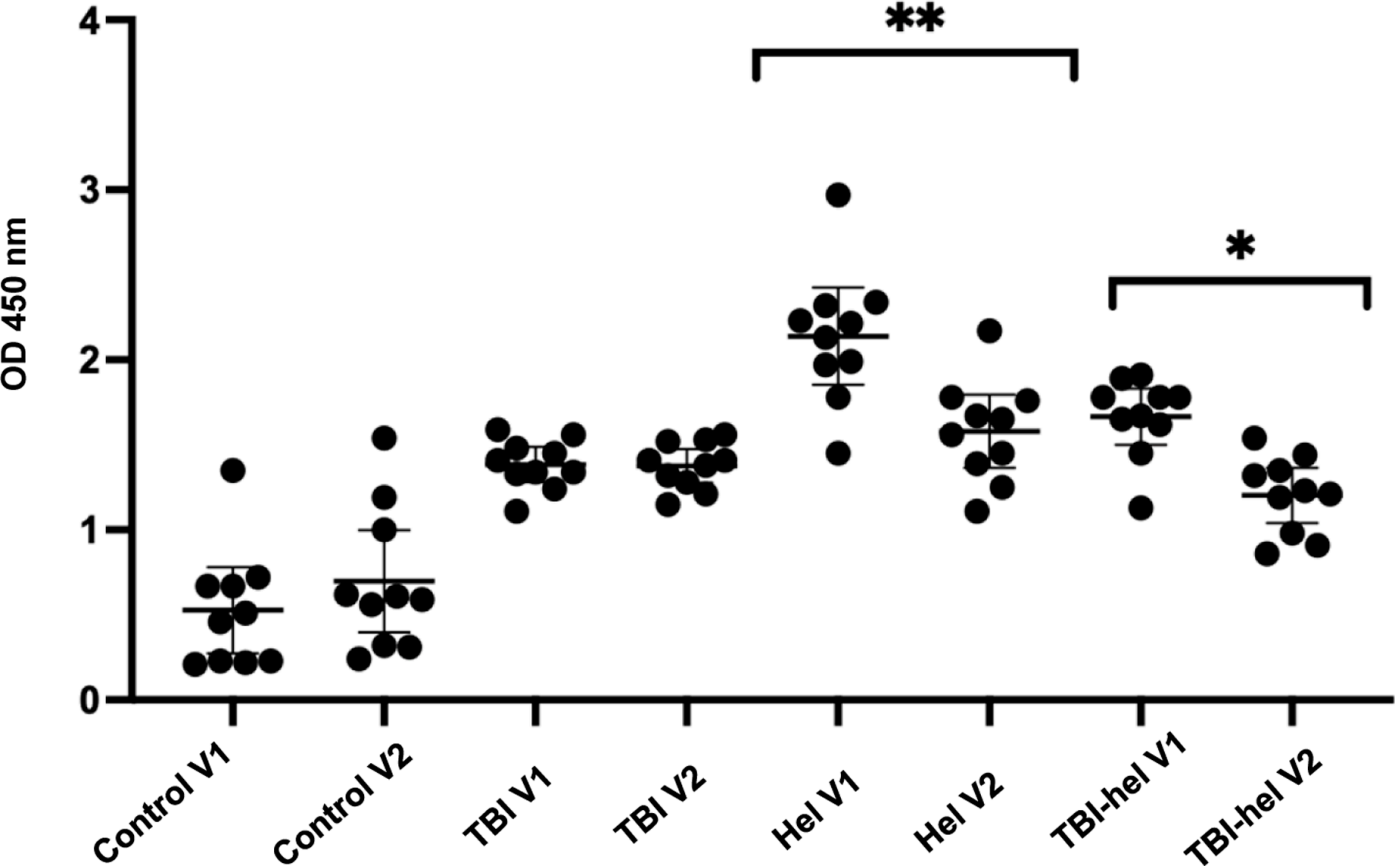
Total IgG4 responses measured using ELISA in controls, TBI, helminth-infected patients and TBI-helminth co-infected patients. Plasma from 10 patients of each group (controls, TBI, helminth infected and TBI-helminth co-infected) were used in the ELISA. Helminth-infected patients were infected with *S. mansoni*. OD values at 450 nm were measured using an ELISA plate reader. Lines represent means with SD. A repeated measures ANOVA was performed, where * represented *P* < 0.05 and ** represented *P* < 0.005. OD = optical density; V1 = Visit 1; TBI = TB infection; ELISA = enzyme-linked immunosorbent assay; SD = standard deviation; ANOVA = analysis of variance.

To quantitate total and parasite-specific IgE and IgG4 antibodies and assess the effect of anti-helminthic treatment, plasma samples from 11 individuals from each group infected with *S. mansoni* were tested by ELISA, both before and after treatment. Total IgE was significantly decreased in helminth-infected and TBI-helminth co-infected individuals after treatment (*P* < 0.01 and *P* < 0.005, respectively). IgG4 responses to *S. mansoni* SEA were also significantly decreased in helminth-infected and TBI-helminth co-infected individuals after anti-helminthic treatment (*P* < 0.01 and *P* < 0.05, respectively) (Figures 1 and 2).

### Mycobacterial growth inhibition in TBI or helminth infection

Pre-treatment, compared to the uninfected controls, there was a significant increase in log CFU in those with TBI (*P* < 0.05), in those infected with helminths (*P* < 0.001), or in those with both TBI and helminth co-infection (*P* < 0.001). Cultures from the co-infected group showed more mycobacterial growth than those with either TBI (*P* < 0.001) or helminth infection alone (*P* < 0.05).

**Figure 3.**
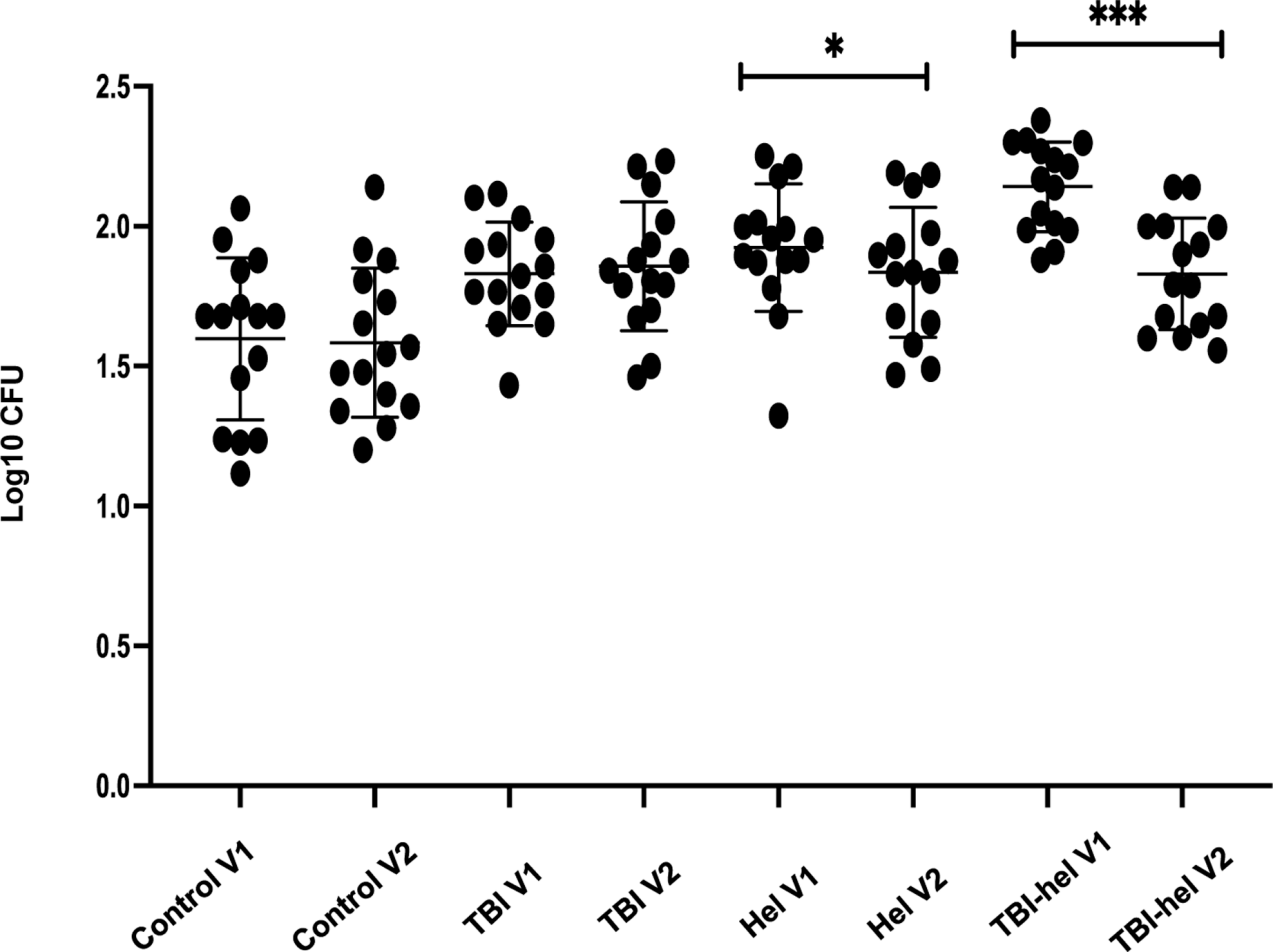
MGIA in controls, TBI, helminth-infected and TBI-helminth-infected patients (V1 and V2). The MGIA assay was performed pre and post anti-helminthic treatment (V1 and V2) in controls, TBI, helminth-infected and TBI-helminth co-infected patients. PBMC from TBI individuals and controls were collected at V1 and V2 (without any treatment). Fifteen donors were tested per group. A two-way ANOVA was performed, where * represented *P* < 0.05 and *** represented a *P* < 0.01. Bars represent the median values with SD. CFU = colony-forming unit; MGIA = mycobacterial growth inhibition assay; V1 = Visit 1; TBI = TB infection; SD = standard deviation; ANOVA = analysis of variance.

### Changes in mycobacterial growth inhibition with anti-helminthic treatment

To assess the effect of treating the helminth infection, paired samples of pre-treatment and post-treatment cryopreserved PBMC for each donor were tested in the same experiment. Mycobacterial growth that had been significantly higher in helminth-infected and TBI helminth-co-infected individuals before treatment was reduced after anti-helminthic treatment (*P* < 0.001). There was no significant change in MGI in controls or those with only TBI at the two visits (Figure 3). TBI-helminth co-infected individuals who had shown the lowest growth inhibition pre-treatment had the greatest improvement in the ability to control the growth of BCG following anti-helminthic treatment.

**Figure 4.**
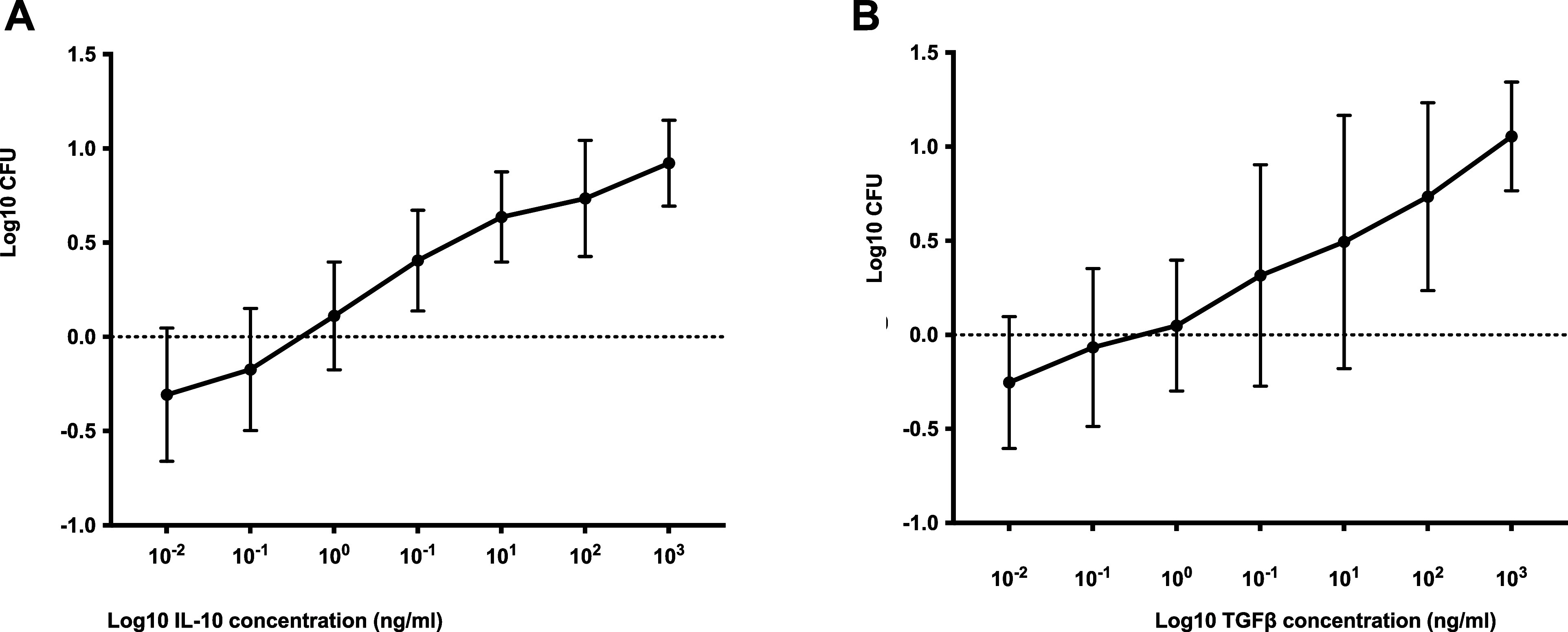
Effect of IL-10 and TGF-ß on mycobacterial growth. The MGIA assay was performed in PBMC collected from three healthy donors, with or without added **A)** rIL-10, or **B)** TGF-ß. Bars represent the median value with SD. There were significant increases in mycobacterial growth with the addition of rIL-10 (*P* < 0.05) and rTGF-ß (Wilcoxon signed rank test) (*P* < 0.05). CFU = colony forming units; IL-10 = interleukin-10; TGF-ß = transforming growth factor-ß; MGIA = mycobacterial growth inhibition assay; PBMC = peripheral blood mononuclear cells; rIL-10 = recombinant IL-10; SD = standard deviation.

MGIA was then performed using both BCG and the *M. tuberculosis* Erdman strain in 10 patients to see whether there were differences in mycobacterial growth inhibition in helminth and TBI-helminth co-infected individuals related to mycobacterial virulence. A significant reduction in mycobacterial growth (increased mycobacterial growth inhibition) was again observed following treatment in helminth-infected individuals (*P* < 0.005) when *M. tuberculosis* Erdman was used. TBI-helminth co-infected individuals had lower mycobacterial growth after helminth treatment when either BCG or *M. tuberculosis* Erdman was used (*P* < 0.01 and *P* < 0.05, respectively).

### Effect of cytokine modulation in the growth inhibition assay

To investigate the role of cytokines in mycobacterial growth inhibition, we selected IL-10 and TGF-β for study as they seem to play the most profound role in suppressing T-cell responses to mycobacterial antigens in the context of TB-helminth co-infection and IFN-γ as helminth infections are also associated with a decreased frequency of CD4+ T-cells secreting IFN-γ. We investigated the role these cytokines played in modulating the ability to control BCG growth by both adding the cytokines directly into the cell cultures and blocking their activity using anti-cytokine antibodies.

**Figure 5.**
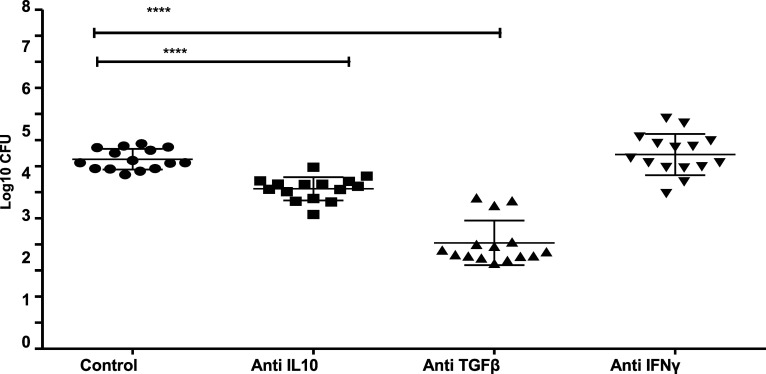
Effect of blocking IL-10, TGF-β, and IFN-γ with specific antibodies on mycobacterial growth. The MGIA assay was performed using frozen PBMC in 15 helminth-infected patients. Anti-IL-10 (6 µg/ml), anti-TGF-β (1 µg/ml) and anti-IFN-γ antibodies (300 ng/ml) were added to the cultures as indicated. Individual data points are shown, with the mean and standard deviation. A two-way ANOVA was performed, followed by Bonferroni multiple test, where ****represented *P* < 0.0001. IL-10 = interleukin-10; TGF-ß = transforming growth factor-ß; IFN-γ = interferon-gamma; MGIA = mycobacterial growth inhibition assay; PBMC = peripheral blood mononuclear cells; ANOVA = analysis of variance.

There was a significant increase in mycobacterial growth in cultures supplemented with increasing concentrations of rIL-10 or rTGF-β (Figure 4). There was no significant change in the extent of mycobacterial growth inhibition when IFN-γ was blocked using anti-IFN-γ antibody. However, when IL-10 or TGF-β were blocked using specific antibodies, there was significantly more growth inhibition (Figure 5), which was greatest when TGF-β was neutralised, supporting the immunoregulatory and suppressive roles of these cytokines.

## DISCUSSION

It was hypothesised that helminth infection might reduce the ability of PBMC to control the growth of mycobacteria and that anti-helminthic treatment in TBI-helminth co-infected migrants living in the United Kingdom might lead to reconstitution of the Th1 response and improve the capacity of cells to control mycobacterial growth.

In the Hospital for Tropical Diseases clinic, *Strongyloides* and *Schistosoma* are the most prevalent helminths identified among individuals investigated for eosinophilia.^10,11^ Many of the patients attending the Hospital for Tropical Diseases clinic and enrolled in the study were identified through a study that demonstrated a prevalence of strongyloidiasis of 33% among migrants attending primary care who had a raised eosinophil count.^11,12^ Total and antigen-specific IgG4 concentrations were higher in helminth and TBI-helminth co-infected individuals and eosinophil counts normalised (in this and other University College London Hospital cohorts treated based on serology), and concentrations of both total and helminth-specific antibodies reduced after anti-helminthic treatment, supporting their classification as helminth infected at V1.^13^ Chronic exposure to TB and co-infection with helminths may skew the immune response to Th2 responses and an IgE and IgG4 antibody response.^14^

Mycobacterial growth was higher (and thus mycobacterial growth inhibition was lower) in helminth-infected and TBI-helminth co-infected individuals than in controls. Helminth infections, in addition to Th2 cytokine responses, can induce suppressive regulatory T-cells (Tregs), which produce inhibitory cytokines such as IL-10 and TGF-β that suppress Th1 type responses and can interfere with effector T-cell activation.^15^ In helminth-infected and TBI-helminth co-infected participants, there was an increase in mycobacterial growth at baseline and, thus, a reduction in the ability to control mycobacterial growth compared to controls, which reversed after anti-helminthic treatment. There was significantly greater mycobacterial growth of *M. tuberculosis* Erdman in helminth-infected patients compared to controls, which reversed after anti-helminthic treatment. Thus, the effects of helminth infection and treatment were observed with both BCG and *M. tuberculosis* Erdman, showing that growth inhibition of BCG can be used as a proxy for virulent *M. tuberculosis*.

We found that blocking the regulatory cytokines IL-10 and TGF-β with specific antibodies enhanced growth inhibition while adding rIL-10 or rTGF-β led to increased bacterial growth. The findings are consistent with the evidence that IL-10 and TGF-β have immunosuppressive activity and may contribute to mycobacterial disease.^15-17^ IL-10 and TGF-β may play an important regulatory role in Strongyloidiasis by upregulating Th9 expression that is reversible upon anti-helminthic treatment, and their ability to suppress mycobacterial growth inhibition shows that these cytokines can inhibit beneficial immune responses to TBI.

MGIA was used here to assess the effect of helminth infection and its treatment on mycobacterial growth inhibition. We showed reduced mycobacterial growth inhibition in those with TBI, as also observed by Lee et al. in South Korea.^18^ The opposite effect was shown in a study in Nepali military recruits with TBI and hookworm infection, with a negative association of MGIA with TBI and reduced MGIA when hookworm infections were treated.^19^ The time course of acquisition of both helminth and TBI is likely to differ between populations, not only between those sampled within and after migration from endemic settings.

Helminth-infected individuals in our study displayed poor growth inhibition in MGIA, which improved after anti-helminthic treatment, indicating that this immunomodulation was helminth-mediated. Helminth infections are, therefore another factor that may adversely affect the ability of the immune system to control mycobacterial infections.^20^ Larger longitudinal studies are now needed to confirm these immunomodulatory effects and their impact in communities where helminth infections and *M. tuberculosis* infections are common, as the numbers studied here were small, which is a limitation of our work. It would also be interesting to investigate the long-term effects of anti-helminthic treatment on TB progression in different populations.

## References

[1] Babu S, Helminth-tuberculosis co-infection: an immunologic perspective. Trends Immunol. 2016;37(9):597–607. 27501916 10.1016/j.it.2016.07.005 PMC5003706

[2] Chen F, An essential role for the Th2-type response in limiting tissue damage during helminth infection. Nat Med. 2012;18(2):260–266. 22245779 10.1038/nm.2628 PMC3274634

[3] Wallis RS, A whole blood bactericidal assay for tuberculosis. J Infect Dis. 2001;183(8):1300–1303. 11262217 10.1086/319679

[4] Hoft DF, Investigation of the relationships between immune-mediated inhibition of mycobacterial growth and other potential surrogate markers of protective *Mycobacterium tuberculosis* immunity. J Infect Dis. 2002;186(10):1448–1457. 12404160 10.1086/344359

[5] Marsay L, Mycobacterial growth inhibition in murine splenocytes as a surrogate for protection against *Mycobacterium tuberculosis* (M. tb). Tuberculosis (Edinb). 2013;93(5):551–557. 23726784 10.1016/j.tube.2013.04.007

[6] Tanner R, Optimisation, harmonisation and standardisation of the direct mycobacterial growth inhibition assay using cryopreserved human peripheral blood mononuclear cells. J Immunol Methods. 2019;469:1–10. 30710562 10.1016/j.jim.2019.01.006 PMC7926177

[7] Araujo Z, Patients exposed to *Mycobacterium tuberculosis* infection with a prominent IgE response. Arch Med Res. 2012;43(3):225–232. 22564424 10.1016/j.arcmed.2012.04.002

[8] Adjobimey T, Induction of immunoglobulin G4 in human filariasis: an indicator of immunoregulation. Ann Trop Med Parasitol. 2010;104(6):455–464. 20863434 10.1179/136485910X12786389891407 PMC3065634

[9] Toulza F, *Mycobacterium tuberculosis*-specific CD4+ T-cell response is increased, and Treg cells decreased, in anthelmintic-treated patients with latent TB. Eur J Immunol. 2016;46(3):752–761. 26638865 10.1002/eji.201545843

[10] Whetham J, Investigation of tropical eosinophilia; assessing a strategy based on geographical area. J Infect. 2003;46(3):180–185. 12643868 10.1053/jinf.2002.1108

[11] Barrett J, The changing aetiology of eosinophilia in migrants and returning travelers in the Hospital for Tropical Diseases, London 2002–2015: an observational study. J Infect. 2017;75(4):301–308. 28842188 10.1016/j.jinf.2017.08.007

[12] Baker EC, High prevalence of Strongyloides among South Asian migrants in primary care—associations with eosinophilia and gastrointestinal symptoms. Pathogens. 2020;9(2):103. 32041352 10.3390/pathogens9020103 PMC7168230

[13] Ming DK, Clinical and diagnostic features of 413 patients treated for imported strongyloidiasis at the hospital for tropical diseases, London. Am J Trop Med Hyg. 2019;101(2):428–431. 31219002 10.4269/ajtmh.19-0087 PMC6685552

[14] Grencis RK. Immunity to helminths: resistance, regulation, and susceptibility to gastrointestinal nematodes. Annu Rev Immunol. 2015;33:201–225. 25533702 10.1146/annurev-immunol-032713-120218

[15] Shevach EM, Control of T-cell activation by CD4+ CD25+ suppressor T cells. Immunol Rev. 2001;182:58–67. 11722623 10.1034/j.1600-065x.2001.1820104.x

[16] Johnston CJ, TGF-β in tolerance, development and regulation of immunity. Cell Immunol. 2016;299:14–22. 26617281 10.1016/j.cellimm.2015.10.006 PMC4711336

[17] Redford PS, The role of IL-10 in immune regulation during *M. tuberculosis* infection. Mucosal Immunol. 2011;4(3):261–270. 21451501 10.1038/mi.2011.7

[18] Lee H, In vitro mycobacterial growth inhibition in South Korean adults with latent TB infection. Front Immunol. 2019;10:896. 31105706 10.3389/fimmu.2019.00896 PMC6497970

[19] O’Shea MK, Human hookworm infection enhances mycobacterial growth inhibition and associates with reduced risk of tuberculosis infection. Front Immunol. 2018;9:2893. 30619265 10.3389/fimmu.2018.02893 PMC6302045

[20] Goletti D, Epidemiology, pathogenesis, clinical presentation and management of TB in patients with HIV and diabetes. Int J Tuberc Lung Dis. 2023;27(4):284–290. 37035976 10.5588/ijtld.22.0685 PMC10094052

